# Diabetes Care in the Psychiatric Inpatient Setting: A National Survey of Mental Health Professionals Knowledge, Attitudes and Skills

**DOI:** 10.1192/bjo.2022.196

**Published:** 2022-06-20

**Authors:** Zoe Goff, Ioana Varvari, Vishal Sharma

**Affiliations:** 1 Leeds and York Partnership Foundation Trust, Leeds, United Kingdom; 2 Tees Esk and Wear Valley NHS Foundation Trust, York, United Kingdom

## Abstract

**Aims:**

People with Severe Mental Illness (SMI) are at increased risk of developing diabetes. There is currently a lack of monitoring and standardisation of diabetes care in the NHS psychiatric inpatient setting. This presents as a missed opportunity, as inpatient admission could be used to improve diabetes care for this population. We surveyed the multi-disciplinary teams in psychiatric inpatient units across England to develop understanding of current diabetes care in this setting.

**Methods:**

A 13-item questionnaire was designed to assess the knowledge, attitudes and skills relating to diabetes care. This was piloted via think out loud interviews with 5 staff at a Forensic unit. Amendments were then made to the questionnaire to improve the validity prior to national roll-out.

Site coordinators working within General Adult, Old Age, Rehabilitation and Forensic inpatient services were recruited via medical education and academic links. This included 19 inpatient sites within 11 NHS Mental Health Trusts across England. Site coordinators circulated the questionnaire, primarily via electronic survey. A small number of paper responses were also collected.

**Results:**

156 responses were collected via the national survey (electronic = 136, paper = 20). 6 responses were excluded due to missing professional role information or roles not involving physical healthcare. Respondents included within the analysis comprised 43 Doctors, 55 Nurses and 52 Allied Healthcare Professionals.

93% of respondents agreed that addressing physical health needs was an important part of the mental health team's role, although only 28% had received physical healthcare training within the last 12 months.

68% agreed that they had adequate skills and knowledge to manage diabetes safely on the ward. 69% agreed that the diabetic care on the ward was of an acceptable standard according to National Institute for Health and Care Excellent (NICE) guidelines. This reflects a need for appropriate training and guidance to help improve this aspect of care.

Additionally, only 51% agreed that they felt able to refer a patient with diabetes to the most appropriate diabetes service based on type of diabetes and medication prescribed. This highlights an important issue, as cohesive shared-care and clear referral pathways are key when considering effective diabetes management.

**Conclusion:**

Psychiatric inpatient admission could be used opportunistically to improve the healthcare disparities for people with comorbid diabetes and SMI. This national survey highlights key areas that would need to be addressed to standardise and optimise diabetes care in this setting. This includes appropriate training, clear guidelines and cohesive shared-care pathways.

